# The economic value of R_0_ for selective breeding against microparasitic diseases

**DOI:** 10.1186/s12711-020-0526-y

**Published:** 2020-01-31

**Authors:** Kasper Janssen, Piter Bijma

**Affiliations:** Animal Breeding and Genomics, Wageningen University, Droevendaalsesteeg 1, 6708 PB Wageningen, The Netherlands

## Abstract

**Background:**

Microparasitic diseases are caused by bacteria and viruses. Genetic improvement of resistance to microparasitic diseases in breeding programs is desirable and should aim at reducing the basic reproduction ratio ${\text{R}}_{0}$. Recently, we developed a method to derive the economic value of ${\text{R}}_{0}$ for macroparasitic diseases. In epidemiological models for microparasitic diseases, an animal’s disease status is treated as infected or not infected, resulting in a definition of ${\text{R}}_{0}$ that differs from that for macroparasitic diseases. Here, we extend the method for the derivation of the economic value of ${\text{R}}_{0}$ to microparasitic diseases.

**Methods:**

When ${\text{R}}_{0} \le 1$, the economic value of ${\text{R}}_{0}$ is zero because the disease is very rare. When ${\text{R}}_{0}$. is higher than 1, genetic improvement of ${\text{R}}_{0}$ can reduce expenditures on vaccination if vaccination induces herd immunity, or it can reduce production losses due to disease. When vaccination is used to achieve herd immunity, expenditures are proportional to the critical vaccination coverage, which decreases with ${\text{R}}_{0}$. The effect of ${\text{R}}_{0}$ on losses is considered separately for epidemic and endemic disease. Losses for epidemic diseases are proportional to the probability and size of major epidemics. Losses for endemic diseases are proportional to the infected fraction of the population at the endemic equilibrium.

**Results:**

When genetic improvement reduces expenditures on vaccination, expenditures decrease with ${\text{R}}_{0}$ at an increasing rate. When genetic improvement reduces losses in epidemic or endemic diseases, losses decrease with ${\text{R}}_{0}$ at an increasing rate. Hence, in all cases, the economic value of ${\text{R}}_{0}$ increases as ${\text{R}}_{0}$ decreases towards 1.

**Discussion:**

${\text{R}}_{0}$ and its economic value are more informative for potential benefits of genetic improvement than heritability estimates for survival after a disease challenge. In livestock, the potential for genetic improvement is small for epidemic microparasitic diseases, where disease control measures limit possibilities for phenotyping. This is not an issue in aquaculture, where controlled challenge tests are performed in dedicated facilities. If genetic evaluations include infectivity, genetic gain in ${\text{R}}_{0}$ can be accelerated but this would require different testing designs.

**Conclusions:**

When ${\text{R}}_{0} \le 1$, its economic value is zero. The economic value of ${\text{R}}_{0}$ is highest at low values of ${\text{R}}_{0}$ and approaches zero at high values of ${\text{R}}_{0}$.

## Background

Microparasitic diseases are diseases that are caused by bacteria and viruses. Genetic improvement of resistance to microparasitic diseases in breeding programs for livestock and aquaculture species is of interest for the same reasons as for macroparasitic diseases, as recently discussed in Janssen et al. [1]. Genetic improvement should aim at reducing the risk and severity of disease outbreaks, which are both determined by the basic reproduction ratio, ${\text{R}}_{0}$ [2, 3]. However, the definition of ${\text{R}}_{0}$ is different for microparasitic diseases than for macroparasitic diseases, because the disease status of animals is treated differently. For most macroparasitic diseases, the number of parasites per host can be counted and the severity of the clinical symptoms increases as the number of parasites increases [4]. Thus, in epidemiological models for macroparasitic diseases, an animal’s disease status is measured by the number of macroparasites that it carries, e.g. [5]. For most microparasitic diseases, the number of parasites per host cannot be recorded. In epidemiological models for microparasitic diseases, an animal’s disease status is treated as infected or not infected, without any differentiation in the degree of infection. Thus, ${\text{R}}_{0}$ is defined as the expected number of secondary cases produced by a typical infected individual in a completely susceptible population, during its entire period of infectiousness [6]. ${\text{R}}_{0}$ has a threshold value of 1, below which a disease is very rare.

We distinguish two types of microparasitic diseases: epidemic and endemic diseases. In epidemic diseases, animals become immune after infection, so that an outbreak can occur only once per production cycle. In endemic diseases, animals do not become immune after infection and can thus be infected multiple times during a production cycle. Therefore, we classify epidemic diseases as those that lead to incidental outbreaks and endemic diseases as those that are virtually always present and for which the infected fraction of the population varies around its endemic equilibrium [7]. For epidemic diseases, the risk and size of major epidemics are determined by ${\text{R}}_{0}$. For endemic diseases, the level of the endemic equilibrium is determined by ${\text{R}}_{0}$. When ${\text{R}}_{0}$ drops below 1, major epidemics cannot occur and a disease cannot become endemic. Thus, breeding programs should aim at reducing ${\text{R}}_{0}$ ultimately to become below 1, at which point the population is no longer affected. Therefore, as for macroparasitic diseases, ${\text{R}}_{0}$ is the appropriate breeding goal trait for both epidemic and endemic microparasitic diseases [2].

The economic value of ${\text{R}}_{0}$ needs to be derived to optimize its relative emphasis in the breeding goal. Recently, Janssen et al. [1] presented a method to derive the economic value of ${\text{R}}_{0}$ for macroparasitic diseases. Here, we extend their method to microparasitic diseases.

## Methods

To derive the economic value of ${\text{R}}_{0}$, the relationship between costs and ${\text{R}}_{0}$ must be known. Costs of a disease are the sum of expenditures (${\text{E}}$) on disease control and production losses (${\text{L}}$) [8]. Depending on the disease management strategy in the population, the economic value of ${\text{R}}_{0}$ can be derived as the partial derivative of ${\text{E}}$ with respect to ${\text{R}}_{0}$, while ${\text{L}}$ is held constant [1]:

1 $${\text{EV}} = \partial {\text{E}}/\partial {\text{R}}_{0} ,$$

or as the partial derivative of ${\text{L}}$. with respect to ${\text{R}}_{0}$ while ${\text{E}}$ is held constant:

2 $${\text{EV}} = \partial {\text{L}}/\partial {\text{R}}_{0} .$$

Because a reduction in ${\text{R}}_{0}$ increases farm profit, its economic value is negative. However, for presentation purposes, we ignore the minus sign in the economic value throughout the remainder of the text. When vaccination is possible, genetic improvement of ${\text{R}}_{0}$ can reduce expenditures on vaccination, while losses remain constant. However, when vaccination is not possible, genetic improvement of ${\text{R}}_{0}$ will mainly reduce losses, while expenditures are largely unaffected. Thus, vaccination is the only expenditure considered in the Methods section of this study. In the Discussion, we elaborate on this assumption.

### Economic value of R_0_ when expenditures are reduced

Vaccination is the economic optimum strategy when the reduction in losses from vaccination exceeds expenditures on vaccination. Vaccination reduces the fraction of susceptible individuals in a population and, thus, reduces the number of secondary cases produced by an infected individual, without changing ${\text{R}}_{0}$. Here, the reproduction ratio after vaccination is denoted by ${\text{R}}_{\text{vacc}}$. The fraction of susceptible individuals decreases as vaccination coverage and vaccine effectiveness increase. Hence, ${\text{R}}_{\text{vacc}}$ decreases as vaccination coverage and vaccine effectiveness increase. Vaccination coverage ($v_{c}$) is the proportion of the population that is vaccinated. Vaccine effectiveness ($e$) can be defined either as the relative reduction in susceptibility of vaccinated animals compared to unvaccinated animals, or as the proportion of vaccinated animals that remains fully susceptible, while the other part is fully resistant. Both definitions of $e$ have the same implications for ${\text{R}}_{\text{vacc}}$. Both $v_{c}$ and $e$ take values between 0 and 1. ${\text{R}}_{\text{vacc}}$ is a function of ${\text{R}}_{0}$, $v_{c}$ and $e$ [9]:

3 $${\text{R}}_{\text{vacc}} = {\text{R}}_{0} \cdot \left( {1 - v_{c} \cdot e} \right).$$

When ${\text{R}}_{\text{vacc}} \le 1$, herd immunity is achieved and no major outbreak can occur. When herd immunity can be achieved, the critical vaccination coverage is the minimum proportion of the population that must be vaccinated in order to induce herd immunity. In the Discussion, we address the effect of uncertainty in the estimate of ${\text{R}}_{0}$ on the critical vaccination coverage. The critical vaccination coverage is the economic optimum vaccination coverage, with lower and higher levels increasing the costs of the disease (results not shown). Because economic values should be derived under optimized management [10], the economic value of ${\text{R}}_{0}$ is derived from the reduction in expenditures on vaccination at the critical vaccination coverage. We assume that expenditures are proportionate to the critical vaccination coverage, i.e. that expenditures per vaccinated animal are constant. The critical vaccination coverage decreases as ${\text{R}}_{0}$ decreases, so expenditures decrease as ${\text{R}}_{0}$ decreases. At the same time, herd immunity sets the losses to zero, so there is no need to differentiate between epidemic and endemic diseases. Thus, the economic value of ${\text{R}}_{0}$ can be derived from a reduction in expenditures on vaccination while losses are kept constant, i.e. when losses are zero. The algebra to define expenditures as a function of ${\text{R}}_{0}$ and for the derivation of the economic value of ${\text{R}}_{0}$ is provided in the following.

${\text{E}}$ is a function of the critical vaccination coverage, population size, and expenditures per vaccinated animal. The critical vaccination coverage ($v_{crit}$) is reached when ${\text{R}}_{\text{vacc}} = 1$. According to [9], rewriting Eq. (3) for ${\text{R}}_{\text{vacc}} = 1$ gives:

4 $$v_{crit} = \left( {1 - 1/{\text{R}}_{0} } \right)/e.$$

$v_{crit}$ increases with ${\text{R}}_{0}$. to an asymptote of $1/e$, and decreases to $1 - 1/{\text{R}}_{0}$ as $e$ approaches 1. Any vaccination coverage that is equal to or higher than $v_{crit}$ induces herd immunity. Let ${\text{N}}$ be the population size and let ${\text{E}}_{\text{vacc}}$ be expenditures per vaccinated animal. Under these assumptions, ${\text{E}}$ is a function of ${\text{R}}_{0}$ as:

5 $${\text{E}} = v_{crit} \cdot {\text{N}} \cdot {\text{E}}_{\text{vacc}} = \left( {1 - 1/{\text{R}}_{0} } \right)/e \cdot {\text{N}} \cdot {\text{E}}_{\text{vacc}} .$$

From Eqs. (1) and (5), it follows that:

6 $${\text{EV}} = \frac{{\partial {\text{E}}}}{{\partial {\text{R}}_{0} }} = \frac{{\partial \left( {\left( {1 - 1/{\text{R}}_{0} } \right)/e \cdot {\text{N}} \cdot {\text{E}}_{\text{vacc}} } \right)}}{{\partial {\text{R}}_{0} }} = \frac{{{\text{N}} \cdot {\text{E}}_{\text{vacc}} }}{e} \cdot \frac{{\partial \left( {1 - 1/{\text{R}}_{0} } \right)}}{{\partial {\text{R}}_{0} }} = \frac{{{\text{N}} \cdot {\text{E}}_{\text{vacc}} }}{{e \cdot {\text{R}}_{0}^{2} }},$$

which gives the economic value when a decrease in ${\text{R}}_{0}$ reduces expenditures on vaccination.

When the critical vaccination coverage exceeds its theoretical maximum value of 1 (Eq. (4)), herd immunity (${\text{R}}_{\text{vacc}} \le 1)$ cannot be achieved. Thus, when ${\text{R}}_{0}$ is high and a vaccine’s effectiveness is low, herd immunity cannot be achieved regardless of the vaccination coverage. In such cases, genetic improvement of ${\text{R}}_{0}$ reduces losses while expenditures are held constant, which is dealt with in the sections below, where ${\text{R}}_{0}$ can be substituted by ${\text{R}}_{\text{vacc}}$ to account for the effect of vaccination.

### Economic value of R_0_ for epidemic diseases when losses are reduced

The simplest model for epidemic diseases is a SIR-model, where animals are classified according to three mutually exclusive states: susceptible, infected, and recovered [11, 12]. In this model, all animals are susceptible before a first epidemic. An epidemic can only occur after infection from an external source. After infection from an external source, susceptible animals can become infected and infected animals can recover. Neither infected nor recovered animals can become susceptible again. Hence, recovered animals have acquired full immunity. The term ‘removed’ is sometimes used instead of ‘recovered’ to indicate that these animals no longer affect the epidemic.

We assume that losses are proportional to the average proportion of the population that gets infected during a production cycle. In other words, we ignore variation in disease tolerance and assume constant losses per infected animal. The average proportion of the population that gets infected during a production cycle is equal to the product of the probability of an epidemic in a production cycle and the expected fraction of the population that gets infected in case of an epidemic. The probability of an epidemic in a production cycle increases with the frequency of infections from an external source. Following infection from an external source, it leads to either a minor or a major epidemic. During a minor epidemic, a negligible fraction of the population gets infected and the epidemic dies out quickly. During a major epidemic, a significant fraction of the population gets infected. Provided that ${\text{R}}_{0} > 1$, the probability for a minor epidemic following infection from an external source is equal to $1/{\text{R}}_{0}$ [12]. Thus, minor epidemics occur even when ${\text{R}}_{0} > 1$. After a minor epidemic, the probability of a (next) epidemic remains virtually unchanged because the susceptible fraction of the population remains largely unaltered. Since relatively few animals get infected during minor epidemics, we ignore the effect of minor epidemics on losses. Infection from an external source results in a major epidemic with probability $11/{\text{R}}_{0}$, i.e. the probability of not resulting in a minor epidemic [12]. The final fraction of the population that has been infected by the end of a major epidemic increases with ${\text{R}}_{0}$, to an asymptote of 1 [12]. The final fraction of the population that is still susceptible after a major epidemic is too small for a second major epidemic to occur. Based on the above, losses can be defined as a function of ${\text{R}}_{0}$, from which the economic value of ${\text{R}}_{0}$ can be derived, as shown below.

${\text{L}}$ is a function of the probability of a major epidemic in a production cycle, the final fraction of the population that has been infected by the end of a major epidemic, population size, and production losses per infected individual. Let $m$ be the frequency of infections from an external source per production cycle. The probability that a single infection from an external source results in a minor epidemic is $1/{\text{R}}_{0}$; hence the probability that all $m$ infections from an external source result in a minor epidemic is $\left( {\frac{1}{{{\text{R}}_{0} }}} \right)^{m}$. Thus, the probability ($p$) of a major epidemic during a production cycle is equal to [12]:

7 $$p\left( {{\text{R}}_{0} } \right)|_{{{\text{R}}_{0} > 1}} = 1 - \left( {\frac{1}{{{\text{R}}_{0} }}} \right)^{m} .$$

**Fig. 1:**
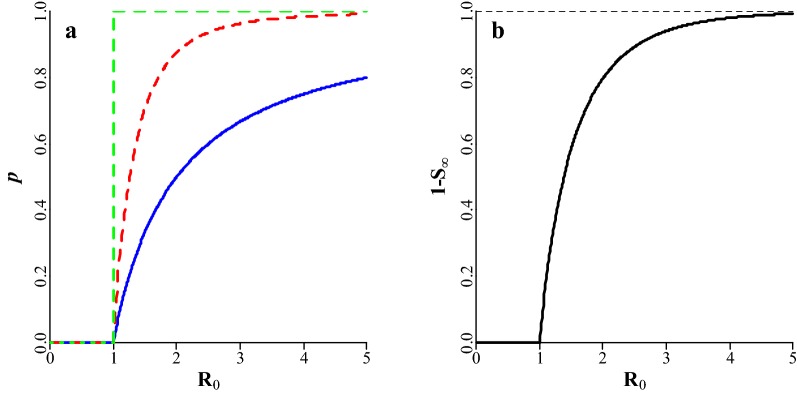
**a** Relationship between ${\text{R}}_{0}$ and the probability (*p*) of a major epidemic during a production cycle for different frequencies of infection from an external source (*m*). Solid blue line: $m = 1$, red dashed line: $m = 3$, green dashed line: $m = \infty$. **b** Relationship between ${\text{R}}_{0}$ and the final fraction of the population that has been infected by the end of a major epidemic ($1 - {\text{S}}_{\infty }$) in epidemic microparasitic diseases. The dashed black line indicates the asymptote

This probability increases with ${\text{R}}_{0}$ and with $m$, to an asymptote of 1 (Fig. 1a). The final fraction of the population that has been infected by the end of a major epidemic is denoted by $1 - {\text{S}}_{\infty }$, where ${\text{S}}_{\infty }$ is the fraction of the population that remains susceptible after the epidemic has ended, i.e. the fraction that escapes infection. Following Britton [12]:

8 $$1 - {\text{S}}_{\infty } = - \ln \left( {{\text{S}}_{\infty } } \right)/{\text{R}}_{0} .$$

This fraction increases with ${\text{R}}_{0} ,$ to an asymptote of 1 (Fig. 1b). Equation (8) has no algebraic solution, but can be solved numerically. Let ${\text{N}}$ be the population size and ${\text{L}}_{\text{ind}}$ the losses per infected animal. In a SIR-model, animals can get infected only once, so that losses can be incurred only once per animal. Under these assumptions, ${\text{L}}$ is a function of ${\text{R}}_{0}$ as:

9 $${\text{L}}\left( {{\text{R}}_{0} } \right) = p\left( {{\text{R}}_{0} } \right) \cdot (1 - {\text{S}}_{\infty } ) \cdot {\text{N}} \cdot {\text{L}}_{\text{ind}} .$$

From Eqs. (2) and (9), it follows that:

10 $$\begin{aligned} {\text{EV}} & = \frac{{\partial {\text{L}}}}{{\partial {\text{R}}_{0} }} = \frac{{\partial \left( {p\left( {{\text{R}}_{0} } \right) \cdot (1 - {\text{S}}_{\infty } ) \cdot {\text{N}} \cdot {\text{L}}_{\text{ind}} } \right)}}{{\partial {\text{R}}_{0} }} \hfill \\ & = {\text{N}} \cdot {\text{L}}_{{{\text{ind}}.}} \cdot \left( {\frac{{\partial p\left( {{\text{R}}_{0} } \right)}}{{\partial {\text{R}}_{0} }} \cdot \left( {1 - {\text{S}}_{\infty } } \right) + \frac{{\partial \left( {1 - {\text{S}}_{\infty } } \right)}}{{\partial {\text{R}}_{0} }} \cdot p\left( {{\text{R}}_{0} } \right)} \right), \hfill \\ \end{aligned}$$

where

11 $$\frac{{\partial p\left( {{\text{R}}_{0} } \right)}}{{\partial {\text{R}}_{0} }} = \frac{m}{{{\text{R}}_{0}^{m + 1} }},$$

and since ${\text{R}}_{0} = - \frac{{\ln \left( {{\text{S}}_{\infty } } \right)}}{{1 - {\text{S}}_{\infty } }}$, based on Eq. (8),

12 $$\frac{{\partial \left( {1 - {\text{S}}_{\infty } } \right)}}{{\partial {\text{R}}_{0} }} = - \frac{{\partial {\text{S}}_{\infty } }}{{\partial {\text{R}}_{0} }} = \frac{ - 1}{{\partial {\text{R}}_{0} /\partial {\text{S}}_{\infty } }} = \frac{ - 1}{{\partial \left( { - \frac{{\ln \left( {{\text{S}}_{\infty } } \right)}}{{1 - {\text{S}}_{\infty } }}} \right)/\partial {\text{S}}_{\infty } }} = \frac{{\left( {{\text{S}}_{\infty } - 1} \right)^{2} }}{{1/{\text{S}}_{\infty } + \ln \left( {{\text{S}}_{\infty } } \right) - 1}}$$

Equation (10) gives the economic value of ${\text{R}}_{0}$. for epidemic diseases when a decrease in ${\text{R}}_{0}$ reduces losses, where the derivative expressions are given in Eqs. (11) and (12), $\left( {1 - {\text{S}}_{\infty } } \right)$ in Eq. (8), and $p\left( {{\text{R}}_{0} } \right)$ in Eq. (7). Equation (10) cannot be simplified further.

### Economic value of R_0_ for endemic diseases when losses are reduced

The simplest model for endemic diseases is a SIS-model, where recovered animals immediately become susceptible again. This model assumes that animals do not acquire immunity, such that the population consists of susceptible and infected animals only. Provided that ${\text{R}}_{0} > 1$ and the disease is present, the fractions of susceptible and infected animals in the population tend towards a dynamic endemic equilibrium. The fraction of infected animals at the endemic equilibrium increases with ${\text{R}}_{0}$, to an asymptote of 1. We assume that losses are proportional to the average fraction of the population that is infected at the endemic equilibrium. The algebra to define losses as a function of ${\text{R}}_{0}$ and to derive the economic value of ${\text{R}}_{0}$ is provided below.

**Fig. 2:**
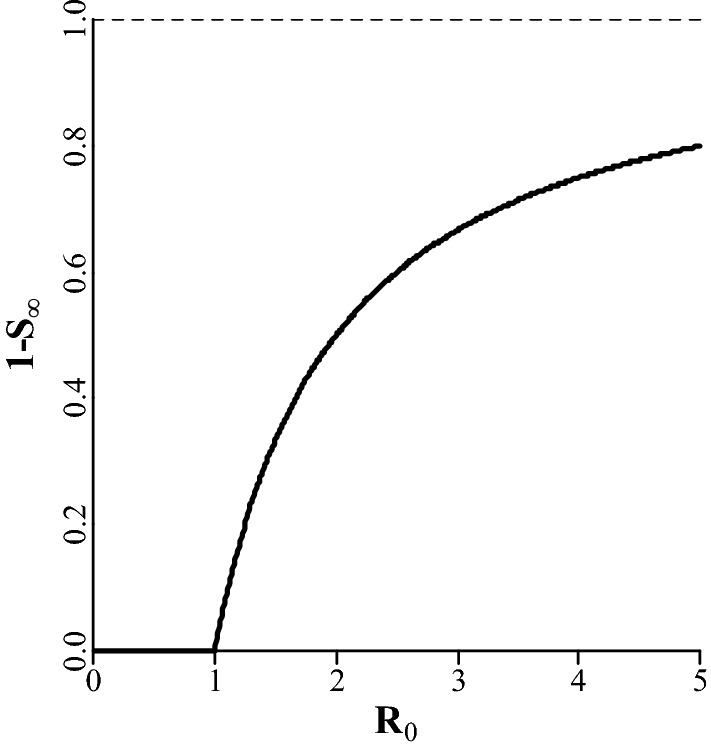
Relationship between ${\text{R}}_{0}$ and the infected fraction of the population ($1 - {\text{S}}_{\infty }$) in endemic microparasitic diseases. The dashed black line indicates the asymptote

${\text{L}}$ is proportional to the fraction of infected animals at the endemic equilibrium, population size, and production losses per infected animal. Let ${\text{S}}_{\infty }$ be the susceptible fraction of the population at the endemic equilibrium, such that $1 - {\text{S}}_{\infty }$ is the infected fraction at the endemic equilibrium. The endemic equilibrium is reached when each infected animal infects on average one other animal before it becomes susceptible again. Hence, ${\text{S}}_{\infty } \cdot {\text{R}}_{0} = 1$, from which it follows that:

13 $$1 - {\text{S}}_{\infty } = 1 - \frac{1}{{{\text{R}}_{0} }},$$

which increases with ${\text{R}}_{0}$, to an asymptote of 1 (Fig. 2) [12]. Let ${\text{N}}$ be again the population size and ${\text{L}}_{\text{ind}}$ the production losses for an individual if it was infected during the entire length of a production cycle. Production losses per day are assumed equal for all infected individuals. Then, ${\text{L}}$ is determined by the average fraction of the population that is infected during the length of a production cycle, regardless of which individuals are infected. Under these assumptions, ${\text{L}}$ is a function of ${\text{R}}_{0}$, as follows:

14 $${\text{L}}\left( {{\text{R}}_{0} } \right) = (1 - {\text{S}}_{\infty } ) \cdot {\text{N}} \cdot {\text{L}}_{\text{ind}} = \left( {1 - \frac{1}{{{\text{R}}_{0} }}} \right) \cdot {\text{N}} \cdot {\text{L}}_{\text{ind}} .$$

From Eqs. (2) and (14), it follows that:

15 $${\text{EV}} = \frac{{\partial {\text{L}}}}{{\partial {\text{R}}_{0} }} = \frac{{\partial \left( {\left( {1 - \frac{1}{{{\text{R}}_{0} }}} \right) \cdot {\text{N}} \cdot {\text{L}}_{\text{ind}} } \right)}}{{\partial {\text{R}}_{0} }} = \frac{{{\text{N}} \cdot {\text{L}}_{\text{ind}} }}{{{\text{R}}_{0}^{2} }},$$

which gives the economic value of ${\text{R}}_{0}$ for endemic diseases when a decrease in ${\text{R}}_{0}$ reduces losses.

## Results

### Economic value of R_0_ when expenditures are reduced

**Fig. 3:**
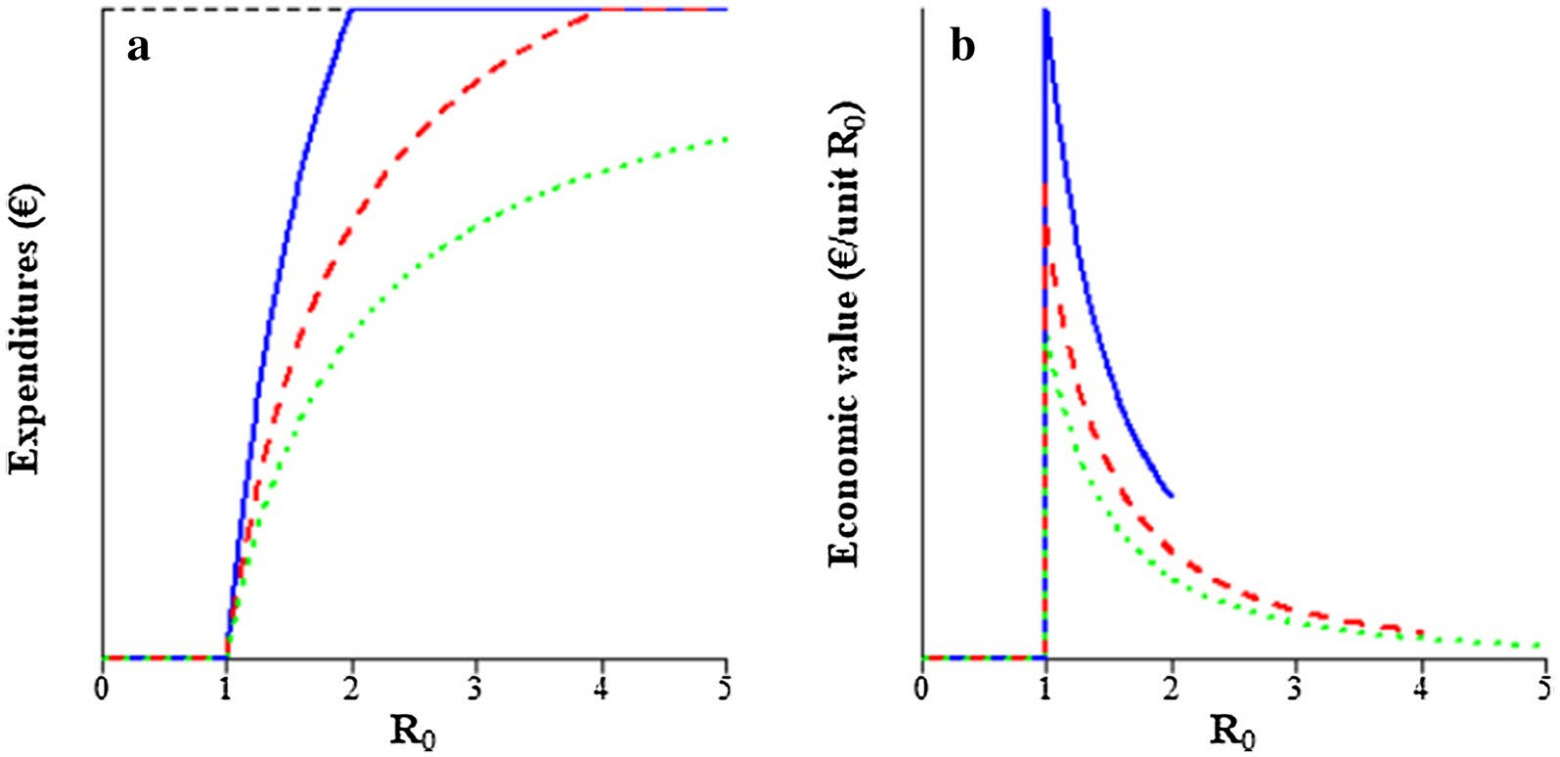
Relationship between ${\text{R}}_{0}$ and **a** expenditures on vaccination (Eq. (5)), and **b** the economic value of ${\text{R}}_{0}$ (Eq. (6)) for microparasitic diseases at different levels of vaccine effectiveness ($e$). The dashed black line in **a** indicates where expenditures are maximum. Solid blue line: $e = 0.5$, dashed red line: $e = 0.75$, dotted green line: $e = 1$. Actual units are omitted from the y-axes because they depend on the number of animals in the population and expenditures per vaccinated animal. Note that the minus sign in the economic value is ignored for presentation purposes

The relationship between ${\text{R}}_{0}$ and expenditures on vaccination and between ${\text{R}}_{0}$ and its economic value are shown in Fig. 3a, b, respectively. Expenditures plateau when the entire population is vaccinated. For values of ${\text{R}}_{0}$ for which expenditures are maximum, herd immunity cannot be attained and the economic value results from a reduction in losses instead. For values of ${\text{R}}_{0}$ below the maximum level of expenditures, herd immunity can be attained and expenditures decrease at an increasing rate when ${\text{R}}_{0}$ decreases. Hence the economic value is relatively high for low values of ${\text{R}}_{0}$.

### Economic value of R_0_ for epidemic diseases when losses are reduced

**Fig. 4:**
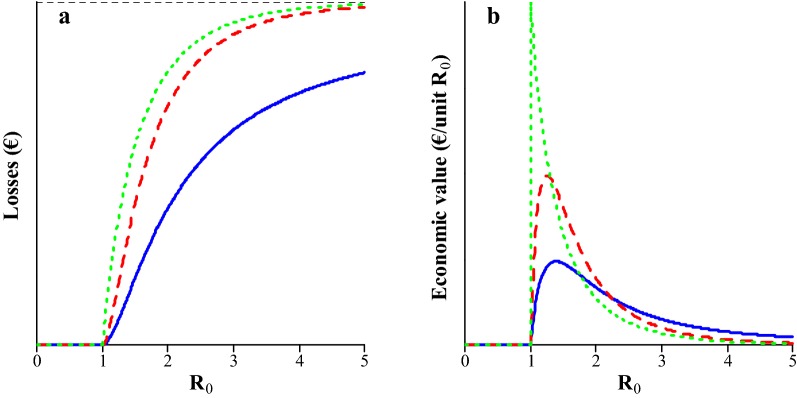
Relationship between ${\text{R}}_{0}$ and **a** losses (Eq. (9)), and **b** the economic value of ${\text{R}}_{0}$ (Eq. (10)) for epidemic microparasitic diseases at different frequencies of infection from an external source ($m$). The dashed black line in **a** indicates the asymptote, i.e. maximum losses. Solid blue line: $m = 1$, dashed red line: $m = 3$, dotted green line: $m = \infty$. Actual units are omitted from the y-axes because they depend on the number of animals in the population and losses per infected animal. Note that the minus sign in the economic value is ignored for presentation purposes

The relationship between ${\text{R}}_{0}$ and losses and between ${\text{R}}_{0}$ and its economic value are shown in Fig. 4a, b, respectively. Losses decrease when ${\text{R}}_{0}$ decreases. At low values of ${\text{R}}_{0}$, losses decrease rapidly with ${\text{R}}_{0}$, resulting in a relatively high economic value. When ${\text{R}}_{0}$ is high and there are frequent infections from an external source, a major epidemic occurs during most production cycles (Fig. 1a), during which virtually the entire population gets infected (Fig. 1b). In such cases, losses are rather insensitive to ${\text{R}}_{0}$, resulting in the economic value being relatively small. Thus, when ${\text{R}}_{0}$ increases to large values, its economic value approaches zero.

For a given value of ${\text{R}}_{0}$, losses increase asymptotically with the frequency of infection from an external source ($m$) (Fig. 4a), because the probability of a major epidemic increases asymptotically with $m$ (Fig. 1a). When ${\text{R}}_{0}$ is low, the probability of a major epidemic increases more rapidly with ${\text{R}}_{0}$ for high than for low values of $m$ (Fig. 1a). As a result, when ${\text{R}}_{0}$ is low, the economic value increases with $m$ (Fig. 4b). When ${\text{R}}_{0}$ is high, the probability of a major epidemic increases more rapidly with ${\text{R}}_{0}$ for low than for high values of $m$ (Fig. 1a). As a result, when ${\text{R}}_{0}$ is high, the economic value decreases with $m$ (Fig. 4b).

### Economic value of R_0_ for endemic diseases when losses are reduced

**Fig. 5:**
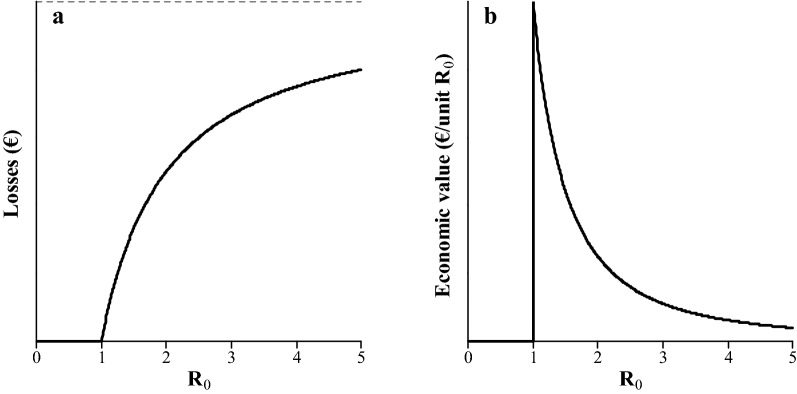
Relationship between ${\text{R}}_{0}$ and **a** production losses (Eq. (14)), and **b** the economic value of ${\text{R}}_{0}$ (Eq. (15)) for endemic microparasitic diseases. The dashed black line in **a** indicates the asymptote, i.e. maximum losses. Actual units are omitted from the y-axes because they depend on the number of animals in the population and losses per infected animal. Note that the minus sign in the economic value is ignored for presentation purposes

The relationship between ${\text{R}}_{0}$ and losses and between ${\text{R}}_{0}$ and its economic value are shown in Fig. 5a, b, respectively. Losses decrease when ${\text{R}}_{0}$ decreases. At low values of ${\text{R}}_{0}$, losses decrease rapidly with ${\text{R}}_{0}$, resulting in a relatively high economic value. When ${\text{R}}_{0}$ is high, virtually the entire population is infected (Fig. 2). In such cases, losses are rather insensitive to ${\text{R}}_{0}$, resulting in a relatively small economic value. Thus, when ${\text{R}}_{0}$ increases to large values, its economic value approaches zero.

## Discussion

This study presents a method to derive the economic value of ${\text{R}}_{0}$ for microparasitic diseases. When ${\text{R}}_{0} \le 1$, its economic value is zero, because the disease is very rare. Otherwise, genetic improvement of ${\text{R}}_{0}$ can reduce expenditures on vaccination if vaccination induces herd immunity, or it can reduce production losses. The critical vaccination coverage decreases with ${\text{R}}_{0}$ (Eq. (4)) and expenditures decrease proportionally (Fig. 3a). The resulting economic value increases as ${\text{R}}_{0}$ decreases towards 1 (Fig. 3b). Both in epidemic and endemic microparasitic diseases, the average fraction of the population that gets infected during a production cycle decreases at an increasing rate when ${\text{R}}_{0}$ decreases towards 1. Resulting losses decrease proportionately (Figs. 4a and 5a). Hence, the economic value of ${\text{R}}_{0}$ is relatively high when ${\text{R}}_{0}$ is low (Figs. 4b and 5b). When ${\text{R}}_{0}$ is high, production losses are relatively insensitive to ${\text{R}}_{0}$, resulting in a relatively low economic value.

### Effect of R_0_ on expenditures and losses

We have shown that genetic improvement of ${\text{R}}_{0}$ can reduce the critical vaccination coverage. However, a prerequisite to reduce critical vaccination coverage is to have reliable estimates of ${\text{R}}_{0}$ and vaccine effectiveness. Methods to estimate ${\text{R}}_{0}$ are in Dietz [9]. Since estimates of ${\text{R}}_{0}$ are sensitive to underlying assumptions, they may be imprecise [9]. Vaccine effectiveness can be evaluated directly from the odds ratio for infection in case–control studies in the population at large, or it may be approximated by the evaluation of vaccine efficacy in controlled conditions [13, 14]. However, vaccine effectiveness is not routinely reported by manufacturers of veterinary vaccines [14]. If the actual value of ${\text{R}}_{0}$ and a vaccine’s effectiveness are uncertain, the critical vaccination coverage and its potential reduction after genetic improvement of ${\text{R}}_{0}$ will also be uncertain. Although Britton [12] provides a method to estimate the standard error of the critical vaccination coverage, this method does not account for uncertainty in vaccine effectiveness. When extra expenditures from vaccinating too many animals compared to the critical vaccination coverage are small relative to potential losses of vaccinating too few animals, a reduction in vaccination coverage after genetic improvement of ${\text{R}}_{0}$ may not be worth the risk. In that case, the economic value of ${\text{R}}_{0}$ will be lower than the theoretical value derived with Eq. (6), or it may even be 0.

Janssen et al. [1] argued that genetic improvement of ${\text{R}}_{0}$ may reduce losses, or expenditures, or both. Here, only losses and expenditures on vaccination were considered, while other expenditures were assumed to be constant. Expenditures on preventive measures, such as fallowing an aquaculture site after each production cycle, are generally attractive control measures because they act against multiple pathogens simultaneously [15]. Therefore, we expect a potential reduction in these expenditures to be lower than the corresponding reduction in production losses, i.e. $\partial {\text{L}}/\partial {\text{E}} < - 1$, such that these expenditures are unlikely to be reduced following genetic improvement. Disease specific preventive measures, including prophylactic treatment, reduce the value of ${\text{R}}_{0}$, while genetic improvement of ${\text{R}}_{0}$ could reduce these expenditures. The outcome of this complex interaction is difficult to predict, but in general we expect little effect of genetic improvement on expenditures for preventive measures [1]. On the other hand, expenditures on curative treatment, e.g., drugs, may be reduced following genetic improvement of ${\text{R}}_{0}$. However, how treatment is applied likely differs between livestock and aquaculture. In livestock, treatment can generally be applied at the level of the individual when infection is detected, e.g., based on clinical signs. Thus, expenditures on treatment of infected animals are proportionate to the average number of infected animals and they can be included in production losses as part of ${\text{L}}_{\text{ind}}$ (Eqs. (10) and (15)). In aquaculture, treatment can only be applied at a group level (cage, tank, pond, etc.). For epidemic diseases, expenditures on treatment are expected to be largely proportional to the probability of major epidemics and can, therefore, decrease as ${\text{R}}_{0}$ decreases (Fig. 1a). For endemic diseases, treatment can temporarily reduce the infected fraction of the population. After treatment, the infected fraction will return to the endemic equilibrium until treatment is applied again. Losses could be assumed proportional to the mean infected fraction over time, while expenditures are proportional to the frequency of treatment. If the frequency of treatment is constant after genetic improvement, losses decrease proportional to the mean infected fraction, while expenditures are constant. If the frequency of treatment decreases after genetic improvement, expenditures decrease and losses remain constant. Such scenarios were evaluated for endemic macroparasitic diseases in Janssen et al. [1]. However, for endemic microparasitic diseases, treatments are not expected to be frequent, because antibiotic usage is strongly restricted by legislation to avoid development of drug-resistant parasites [16].

### Potential for genetic improvement

The results of this study indicate that ${\text{R}}_{0}$ is a useful indicator of the potential economic impact of genetic improvement. Without consideration of the dynamics of disease transmission, one cannot predict the result of selection. For example, Cock et al. [17] reported on ‘success and failure’ of breeding for disease resistance in shrimp. They describe the results of selection for improved resistance against the Taura syndrome virus (TSV) and white spot syndrome virus (WSSV). Before selection, survival after a major outbreak was about 50% for TSV and 2% for WSSV. Assuming that infection leads to death, these survival rates suggest values for ${\text{R}}_{0}$ of about 1.4 for TSV and 4 for WSSV (Eq. (8)). A few generations of selection for resistance to TSV returned survival to levels similar to those before the disease was introduced, while selection for resistance to WSSV had disappointing results. Selection procedures for both diseases were similar and the authors of this study had difficulties to explain these apparently contrasting results. However, given the above estimates of ${\text{R}}_{0}$, such results are not surprising. The low value of ${\text{R}}_{0}$ for TSV suggests that survival after a major epidemic can be improved rapidly, while the higher value of ${\text{R}}_{0}$ for WSSV suggests that improvement is expected to occur at a much slower pace (Fig. 1b). For the above estimates of ${\text{R}}_{0}$, a 30% improvement would be sufficient to eradicate TSV, while the same improvement would increase survival after a major epidemic of WSSV to only 7.5%. This example illustrates that an estimate of heritability is not sufficient to evaluate the potential for economic gain of selection for resistance to a specific disease, because it ignores that resistant animals no longer infect other animals [2]. An estimate of ${\text{R}}_{0}$, ideally combined with its economic value, is at least as important. Although there are numerous estimates of heritability for resistance to specific diseases in aquaculture [18, 19], ${\text{R}}_{0}$ and its economic value are rarely considered. When ${\text{R}}_{0}$ is low, its economic value is likely high, whereas when ${\text{R}}_{0}$ is high, its economic value is likely low (Figs. 3b, 4b and 5b). Thus, even in the absence of genetic parameters, ${\text{R}}_{0}$ and its economic value can be used as the first criteria to judge the potential benefits of selection for resistance to a specific disease. However, in the literature, estimates of ${\text{R}}_{0}$ vary considerably. For example, estimates of ${\text{R}}_{0}$ for WSSV vary from 1.51 [20] to 93 [21], which makes direct interpretation impossible. Thus, consensus and standardization on estimation of ${\text{R}}_{0}$ in epidemiology are required.

### Use of epidemiological models

Previous studies on livestock have demonstrated the need to account for the dynamics of disease transmission to predict the response to selection. For example, for an epidemic microparasitic disease in pigs, MacKenzie and Bishop [22] predicted that the final fraction of the population that will be infected by the end of a major epidemic decreases at an increasing rate with decreasing values of ${\text{R}}_{0}$, just as in Fig. 1b. Similarly, Nieuwhof et al. [23] predicted a substantially higher response in prevalence for an endemic microparasitic disease in sheep when using epidemiological models than when using prevalence as a predictor in a threshold model. These studies demonstrate the need to account for the dynamics of disease transmission in the derivation of the economic value of disease resistance, as also discussed in Janssen et al. [1].

The models used in this study describe within-farm dynamics of disease transmission. However, genetic improvement of ${\text{R}}_{0}$ may also affect the dynamics of disease transmission between farms. In Eq. (7), the probability of a major epidemic during a production cycle is a function of ${\text{R}}_{0}$ and the frequency of infection from an external source, which was assumed to be constant. The source of these infections can be the natural environment or neighbouring farms. Thus, when ${\text{R}}_{0}$ in neighbouring farms improves, disease prevalence on these farms may decrease, reducing the frequency of infection from an external source. Consequently, the probability of a major epidemic would decrease more rapidly after genetic improvement of ${\text{R}}_{0}$, which would increase its economic value. To adequately account for the effect of ${\text{R}}_{0}$ on the frequency of infection from an external source, a combined within- and between-farm epidemiological model would be required, e.g., as in Aldrin et al. [24].

For aquatic environments, it has been questioned to what extent traditional epidemiological models are valid, including the SIR and SIS-models used here. These models have been largely developed for terrestrial populations, where disease transmission occurs on a relatively small spatial scale. In an aquatic environment, disease transmission can occur without direct contact between animals since pathogens can spread over relatively large distances in the water column [25, 26]. Disease dynamics on large spatial scales such as those between farms, may, therefore, be fundamentally different in aquaculture than in livestock. However, for within-farm disease dynamics, indirect transmission via the water column can be accounted for in the transmission rate parameter [20] of the SIR and SIS models.

### Genetic evaluation of R_0_

In livestock, drastic control measures are taken at a regional level as soon as some epidemic microparasitic diseases, such as classical swine fever and foot-and-mouth disease, are detected [27]. Since such control strategies limit routine phenotyping options in breeding programs [28], genetic improvement of ${\text{R}}_{0}$ is not feasible for many epidemic microparasitic diseases in livestock. For endemic diseases, longitudinal data on the disease status of individual animals in farms can sometimes be used. For example, Biemans, et al. [29] estimated breeding values for ${\text{R}}_{0}$ for digital dermatitis from longitudinal data on cows in 12 dairy farms.

In aquaculture, phenotyping for microparasitic diseases is not an issue when controlled challenge tests can be performed in dedicated facilities. In a challenge test, naive fish are exposed to the pathogen via submersion, injection or cohabitation with infected ‘donor’ fish. After some incubation time, phenotypes are recorded as dead or alive. Dead fish are considered susceptible and surviving fish resistant [19]. To maximize phenotypic variation, the endpoint of the challenge test is sometimes chosen such that the survival rate is 50% [30]. There are, however, two main issues with this dead-or-alive phenotype. First, incomplete exposure obscures the phenotypic expression of susceptibility [19]. In any epidemic, some animals may not get infected because they escaped infection due to chance, rather than because they were less susceptible. When the endpoint of the challenge test is set retrospectively to the point at which the survival rate was 50%, the number of obscured phenotypes increases. Second, with a dead-or-alive phenotype, susceptibility and tolerance are confounded, while only susceptibility is of interest when the objective is to improve ${\text{R}}_{0}$. Thus, analyses of binomial survival in threshold models can provide inadequate proxies of susceptibility [31].

Current challenge tests in aquaculture, as described above, capture genetic variation in susceptibility at best, leaving genetic variation in infectivity unexploited. Since susceptibility and infectivity have multiplicative effects on ${\text{R}}_{0}$, genetic gain in ${\text{R}}_{0}$ can be accelerated when both susceptibility and infectivity are included in genetic evaluations [2, 32, 33]. Anche et al. [2] have described how estimated breeding values (EBV) for susceptibility and infectivity can be combined into a single EBV for ${\text{R}}_{0}$. Furthermore, the accuracy of EBV for susceptibility improves when infectivity is included in the model for genetic evaluation [34, 35]. The inclusion of infectivity in genetic evaluation requires testing designs that differ from the currently used challenge tests, as discussed in the following.

The same testing design can be used for epidemic and endemic diseases, because the objective is genetic evaluation of ${\text{R}}_{0}$ rather than a characterisation of the disease. To capture genetic variation in infectivity, some individuals should be infected (and, therefore, become infectious) while others are susceptible. Thus, at the start of a challenge test, only some animals should be artificially infected, after which the disease is allowed to spread naturally [34]. Then, the disease status of each animal, infected or not, must be recorded over the course of the epidemic. Although longitudinal data are required, the sampling frequency does not need to be high, because infection times can be predicted from relatively few sample points [34]. For a fixed total number of samplings, Biemans et al. [29] found that the optimum interval between samplings lies between 25 and 50% of the duration of the infectious period. Multiple epidemic groups are required, with related individuals distributed between groups [2], although the optimal group structure has not yet been studied [29]. For a given testing capacity, having fewer larger groups favours the estimation of susceptibility, whereas having many smaller groups tends to produce better estimates of infectivity [34]. For a SIS model, prediction accuracies for additive genetic effects and heritability estimates improve as disease prevalence increases [34], but this has not been studied for a SIR model. Recently, an experimental design that followed the above considerations was used for the genetic evaluation of susceptibility and infectivity for a macroparasitic disease [36]. This study successfully demonstrated presence of genetic variation in infectivity but did not obtain estimates of genetic parameters and breeding values.

## Conclusions

This study presents a method to derive the economic value of ${\text{R}}_{0}$ for microparasitic diseases. When ${\text{R}}_{0} \le 1$, the economic value is zero, because the disease is very rare. When genetic improvement of ${\text{R}}_{0}$ reduces expenditures on vaccination, the economic value increases as ${\text{R}}_{0}$ decreases towards 1. The effect of ${\text{R}}_{0}$ on production losses differs between epidemic and endemic microparasitic diseases. Nevertheless, when genetic improvement of ${\text{R}}_{0}$ reduces losses, the economic value is highest at low values of ${\text{R}}_{0}$ for both epidemic and endemic microparasitic diseases, while it approaches 0 at high values of ${\text{R}}_{0}$.

## Data Availability

Data sharing is not applicable to this article since no datasets were generated or analysed during the current study.
